# Retrospective cohort analysis comparing changes in blood glucose level and body composition according to changes in thyroid‐stimulating hormone level

**DOI:** 10.1111/1753-0407.13315

**Published:** 2022-09-16

**Authors:** Hyunah Kim, Da Young Jung, Seung‐Hwan Lee, Jae‐Hyoung Cho, Hyeon Woo Yim, Hun‐Sung Kim

**Affiliations:** ^1^ College of Pharmacy Sookmyung Women's University Seoul Republic of Korea; ^2^ Department of Biostatistics, Clinical Research Coordinating Center, Catholic Medical Center The Catholic University of Korea Seoul Republic of Korea; ^3^ Division of Endocrinology and Metabolism, Department of Internal Medicine, Seoul St. Mary's Hospital, College of Medicine The Catholic University of Korea Seoul Republic of Korea; ^4^ Department of Preventive Medicine, College of Medicine The Catholic University of Korea Seoul Republic of Korea; ^5^ Department of Medical Informatics, College of Medicine The Catholic University of Korea Seoul Republic of Korea

**Keywords:** diabetes mellitus, euthyroid, glucose, HbA1c, hypothyroidism, TSH, 糖尿病, 甲状腺功能正常, 血糖, 糖化血红蛋白, 甲状腺功能减退, 促甲状腺激素

## Abstract

**Background:**

In the euthyroid state, the risk of developing diabetes according to changes in thyroid‐stimulating hormone (TSH) levels remains controversial. Additionally, the correlation of various body indices affecting blood glucose levels according to changes in TSH levels over a certain period is not well known.

**Methods:**

Patients who underwent health check‐ups twice at a 2 year interval at a tertiary university hospital between 2009 and 2018 were included. By dividing baseline TSH levels into quartiles (TSH_Q1, Q2, Q3, and Q4), various variables were compared, and their changes after 2 years (∆TSH_Q1, Q2, Q3, and Q4) were confirmed.

**Results:**

Among 15 557 patients, the incidence of diabetes mellitus after 2 years was 2.4% (377/15 557 patients). There was no statistically significant difference in the incidence of diabetes according to TSH_Q (*p* = 0.243) or ∆TSH_Q (*p* = 0.131). However, as TSH levels increased, skeletal muscle mass decreased (*p* < 0.001), and body fat mass and percent body fat significantly increased (*p* < 0.001). As ∆TSH increased, ∆fasting blood glucose and ∆body mass index also significantly increased (all *p* < 0.001). The incidence of diabetes decreased significantly as skeletal muscle mass increased (odds ratio 0.734, *p* < 0.001).

**Conclusions:**

Owing to the short study period, it was not possible to prove a statistical relationship between the incidence of diabetes mellitus and TSH levels in the euthyroid state. Significant decreases in skeletal muscle mass and increases in body mass index and body fat mass according to baseline TSH levels were demonstrated. Therefore, a focus on improving physical functions, such as increasing muscle mass and decreasing fat, is required in this case.

## Graphical abstract

**Highlights**

- Body composition changes were identified by baseline thyroid‐stimulating hormone (TSH) and difference in TSH (∆TSH) levels.
- A positive correlation between ∆TSH and ∆glucose was established.
- Patients with high TSH levels in the euthyroid state need muscle and fat management.

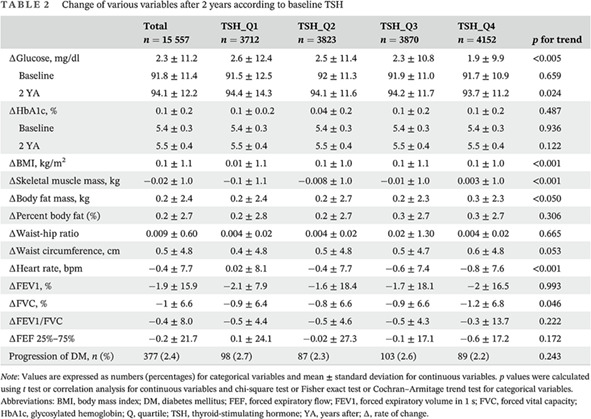

## 摘要

**背景:**

在甲状腺功能正常的状态下，促甲状腺激素(TSH)水平的变化导致糖尿病的风险仍然存在争议。此外，根据TSH水平在一定时期内的变化来影响血糖水平的各种身体指数之间的相关性还不是很清楚。

**方法:**

纳入2009‐2018年间在某三级大学医院接受两次健康检查的患者，每隔两年进行一次健康检查。通过将基线TSH水平分为四个分位数(TSH_Q1, Q2, Q3和Q4)，比较各个变量，并确认它们在两年后的变化(∆TSH_Q1, Q2, Q3和Q4)。

**结果:**

15557例患者中，两年后糖尿病的发生率为2.4%(377/15557例)。根据TSH_Q(p=0.243)或∆TSH_Q(p=0.131)，糖尿病的发病率无统计学差异。但是随着TSH水平的升高，骨骼肌质量下降(p<0.001)，体脂质量和体脂百分比显著增加(p<0.001)。随着∆TSH的升高，∆空腹血糖和∆体重指数也显著升高(均P<0.001)。糖尿病的发病率随着骨骼肌量的增加而显著降低(优势比=0.734，p<0.001)。

**结论:**

由于研究时间较短，尚不能证明糖尿病的发病率与甲状腺功能正常时TSH水平之间的统计关系。与基线TSH水平相比，骨骼肌质量显著减少，身体质量指数和身体脂肪质量增加。因此，在这种情况下，需要把重点放在改善身体功能上，如增加肌肉质量和减少脂肪。

## 1 INTRODUCTION

Thyroid‐stimulating hormone (TSH) is secreted by the pituitary gland and plays an important role in human metabolism by stimulating the production and secretion of thyroid hormones.^1^, ^2^ There are some reports that thyroid hormones increase blood glucose levels through glucose reabsorption, gluconeogenesis, and glycogenolysis^3^; however, there are many opposing opinions that TSH does not affect the actual blood glucose level, as the degree of increase is too weak.^4^

Various studies have shown that the incidence of diabetes is increased in patients with hypothyroidism or hyperthyroidism.^5^, ^6^ In addition, the fasting blood glucose level in patients with subclinical hypothyroidism is higher than that in patients with normal thyroid function.^7^ Conversely, blood glucose levels may also affect thyroid dysfunction.^8^ Changes in thyroid hormones are known to affect blood glucose levels and glycosylated hemoglobin (HbA1c) levels.^9^, ^10^ However, the mechanism underlying the development of diabetes‐related thyroid function is not yet clear and is not well understood. In particular, the understanding of the change in blood glucose according to TSH levels in patients with normal thyroid function remains weak.

Although genetic predisposition plays a major role in diabetes, it is also caused by various personal lifestyle choices, such as a bad diet and low physical activity. Many people are interested in predicting the future risk of diabetes and seek various methods to prevent it. If the risk of diabetes can be predicted using TSH for one medical check‐up or the rate of change in TSH (∆TSH) for two medical check‐ups, patients will have the opportunity to try to increase their skeletal muscle mass and reduce body fat mass.

The higher the TSH level in normal thyroid function, the higher the risk of diabetes.^11^, ^12^ However, various confounding variables were not controlled. A study predicting the risk of diabetes from the TSH levels in adults with normal thyroid function has been conducted in Asia.^13^ In addition, to the best of our knowledge, the relationship between the change in the incidence of diabetes and the rate of ∆TSH rather than the value of TSH itself has not been reported. Studies identifying changes in body composition according to baseline TSH and ∆TSH levels in euthyroid patients are limited. As such, there is a lot of controversy regarding hypothyroidism, but little is known about how TSH levels affect blood glucose itself, especially in the euthyroid state. We hypothesized that the body composition, such as skeletal muscle mass or body fat mass, would change first before blood glucose changed according to TSH or ∆TSH. If this hypothesis is confirmed, patients may be able to engage in various physical activities to change their body composition and prevent diabetes. Therefore, this study aimed to investigate the difference in blood glucose levels according to TSH levels in the euthyroid state in Korea by using health examination data. This can minimize confounding variables because several variables are included, such as the patient's questionnaire, past/current medical history, body composition index, and laboratory tests. In addition, we evaluated whether changes in blood glucose levels and body composition differed according to changes in TSH over 2 years.

## 2 METHODS

### 2.1 Study population and design

From March 2009 to October 2018, patients aged ≥ 18 years who underwent health check‐ups at least twice with an average interval of 2 years (18–30 months) at Seoul St. Mary's Hospital were included. Patients diagnosed with hyperthyroidism/subclinical hyperthyroidism, hypothyroidism/subclinical hypothyroidism, prediabetes, or diabetes at the first medical examination were excluded from this study. Hyperthyroidism/subclinical hyperthyroidism and hypothyroidism/subclinical hypothyroidism were defined by confirming the diagnosis on the patient's questionnaire, verifying the patient's medication, or checking the TSH (reference 0.55–4.78 uIU/ml) and free T4 (reference 0.89–1.76 ng/dl) levels in the health check‐up examination result. Diabetes mellitus was defined by confirming the diagnosis on the patient's questionnaire, verifying the patient's medication, or checking fasting glucose or HbA1c levels.

### 2.2 Differences of variables according to quartile of baseline TSH

**FIGURE 1:**
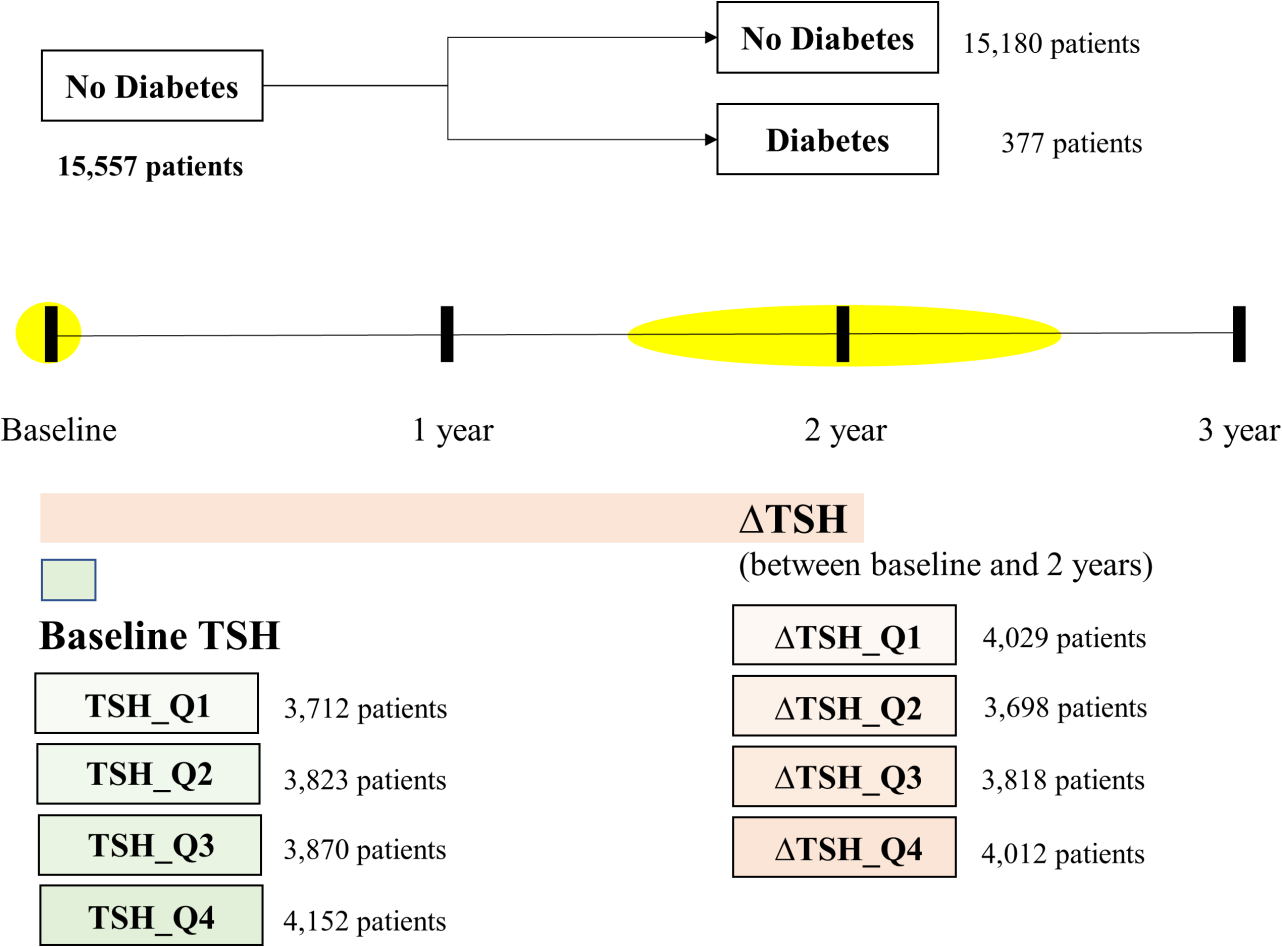
Flowchart of study design

Baseline TSH values measured at the first visit were divided into quartiles (TSH_Q1, TSH_Q2, TSH_Q3, and TSH_Q4), and differences in various variables, such as fasting blood glucose and HbA1c, were confirmed (Figure 1). After 2 years, the rate of change (∆) of each variable according to TSH_Q was checked and compared. The variables used in this study consisted of basic and baseline body information. The basic information included age, sex, height, and weight. Baseline body information, including body mass index (BMI), skeletal muscle mass, body fat mass, percent body fat, and waist‐hip ratio, was measured according to the bioelectrical impedance method using In Body 720 (Bio space, Seoul, Korea). Systolic blood pressure, diastolic blood pressure, and heart rate were measured. Laboratory findings, such as fasting glucose, HbA1c, free T4, blood urea nitrogen (BUN), creatinine, total cholesterol, triglyceride, high‐density lipoprotein cholesterol (HDL‐C), low‐density lipoprotein cholesterol (LDL‐C), creatine phosphokinase (CPK), hemoglobin, hematocrit, aspartate aminotransferase (AST), alanine aminotransferase (ALT), and amylase levels, were collected. Pulmonary function tests, including forced expiratory volume in 1 s (FEV1), forced vital capacity (FVC), FEV1/FVC, and forced expiratory flow 25%–75% (FEF 25%–75%), were carried out. In addition, past medical history of hypertension, dyslipidemia, bronchial asthma, and a family history of diabetes were confirmed.

### 2.3 Differences of variables according to quartile of change of TSH level

After confirming the difference in the change between baseline and second‐visit TSH levels, the difference was also divided into quartiles (∆TSH_Q1, ∆TSH_Q2, ∆TSH_Q3, and ∆TSH_Q4). Variables showing significant findings in baseline TSH were extracted separately, and changes in various variables, such as fasting blood glucose and HbA1c levels, were confirmed. In addition, the number of patients who progressed to diabetes for each ∆TSH level was determined.

### 2.4 Variables affecting the occurrence of diabetes

At the second visit, in those who developed diabetes, we determined whether baseline TSH or ∆TSH levels affected the development of diabetes. The odds ratios (OR) that affected the occurrence of diabetes were calculated using various measured variables. The variables of the second visit were used because there were patients who did not have diabetes at baseline but had developed diabetes at the second visit.

### 2.5 Protection of privacy

This was a retrospective cohort study using data stored in electronic medical records. During the data extraction process, the patient's identifiable information was deleted. All the files for the extracted data were encrypted, and only the corresponding author could access the data used in this study. When the data of this study were delivered to the analyst, a temporary number was assigned and transmitted in an encrypted state. Because this study was data encrypted and anonymized, there was no possibility of mental or physical risk to the subject, and it did not affect the rights and welfare of the subject. Therefore, the patient's consent was not required. Our study was approved by the institutional review board of the Catholic University of Korea, and all guidelines were followed (KC22RISI0044).

### 2.6 Statistical analyses

Categorical variables are presented as numbers (percentages), and continuous variables are presented as mean ± standard deviation. The patients were grouped into quartiles based on their initial TSH levels. *p* values were calculated using the *t* test for continuous variables and the chi‐square test or Fisher's exact test for categorical variables. OR were calculated using logistic regression analysis. All statistical analyses were performed using SAS (version 9.4, SAS Institute, Cary, North Carolina), and *p* < 0.05 was considered statistically significant.

## 3 RESULTS

A total of 15,557 patients who visited Seoul St. Mary's Hospital and underwent health check‐ups at least twice, with an average interval of 2 years, were included in this study. The average age of the patients was 46.7 ± 10.3 years, and 57.5% were male (8941/15,557 patients). The mean BMI was 23.5 ± 3.2 kg/m^2^. The mean fasting glucose level was 91.8 ± 11.4 mg/dl, and the mean HbA1c was 5.4 ± 0.3%; 29.7% of patients (4627/15,557) had a family history of diabetes.

TSH_Q1 comprised 3712 patients (baseline TSH <1.184 uIU/ml), TSH_Q2 comprised 3823 patients (1.184 uIU/ml ≤ baseline TSH < 1.740 uIU/ml), TSH_Q3 comprised 3870 patients (1.740 uIU/ml ≤ baseline TSH < 2.552 uIU/ml), and TSH_Q4 comprised 4152 patients (2.552 uIU/ml ≤ baseline TSH) (Table 1). As TSH levels increased, age also increased significantly (*p* < 0.001). Moreover, as TSH levels increased, there was no significant difference in BMI (*p* = 0.160). Further, the proportion of men was significantly lower (*p* < 0.001) in cases of increased TSH levels. Skeletal muscle mass decreased significantly as TSH levels increased (*p* < 0.001), and body fat mass and percent body fat significantly increased (*p* < 0.001). In addition, both free T4 and heart rate decreased significantly (*p* < 0.001). With the increase in TSH levels, both FVC and FEV1 increased significantly (all *p* < 0.001), and FEV1/FVC also increased, but the increase was not statistically significant (*p* = 0.219).

**TABLE 1:** Baseline characteristics by groups according to baseline TSH level

	Total	TSH_Q1	TSH_Q2	TSH_Q3	TSH_Q4	*p* for trend
	(*n* = 15 557)	(*n* = 3712)	(*n* = 3823)	(*n* = 3870)	(*n* = 4152)	
Age, year	46.7 ± 10.3	46.2 ± 10.1	46.4 ± 10.4	46.6 ± 10.2	47.6 ± 10.6	<0.001
Male sex, *n* (%)	8941 (57.5)	2336 (62.9)	2364 (61.8)	2179 (56.3)	2062 (49.7)	<0.001
Height, cm	166.7 ± 8.6	167.3 ± 9.1	167.4 ± 8.2	166.7 ± 8.2	165.4 ± 8.6	<0.001
BMI, kg/m^2^	23.5 ± 3.2	23.5 ± 3.1	23.6 ± 3.1	23.5 ± 3.1	23.4 ± 3.3	0.160
Skeletal muscle mass, kg	26.8 ± 6.1	27.3 ± 6.1	27.3 ± 6.1	26.8 ± 6.1	25.9 ± 6.2	<0.001
Body fat mass, kg	17.2 ± 5.4	16.9 ± 5.3	17.2 ± 5.3	17.4 ± 5.4	17.4 ± 5.5	<0.001
Percent body fat (%)	26.3 ± 6.3	25.6 ± 6.2	25.8 ± 6.1	26.5 ± 6.2	27.0 ± 6.4	<0.001
Waist‐hip ratio	0.9 ± 0.04	0.9 ± 0.04	0.9 ± 0.04	0.9 ± 0.04	0.9 ± 0.04	<0.050
Waist circumference, cm	83.3 ± 8.7	83.3 ± 8.6	83.6 ± 8.5	83.5 ± 8.5	83.1 ± 9.0	0.227
Systolic BP, mm Hg	119.6 ± 13.7	119.5 ± 13.4	119.7 ± 14	119.7 ± 13.9	119.5 ± 13.7	0.894
Diastolic BP, mm Hg	72.9 ± 10.2	72.8 ± 10.2	73.1 ± 10.3	72.9 ± 10.2	72.7 ± 10.2	0.476
Heart rate, bpm	64.9 ± 9.4	65.2 ± 9.5	65.0 ± 9.5	64.8 ± 9.3	64.5 ± 9.3	<0.001
Laboratory test						
Glucose, mg/dl	91.8 ± 11.4	91.6 ± 10.9	92.3 ± 11.1	91.9 ± 11.1	91.4 ± 12.5	0.660
HbA1c, %	5.4 ± 0.3	5.4 ± 0.3	5.4 ± 0.3	5.4 ± 0.3	5.4 ± 0.3	0.936
free T4, ng/dl	1.3 ± 0.2	1.3 ± 0.2	1.3 ± 0.2	1.2 ± 0.2	1.2 ± 0.2	<0.001
BUN, mg/dl	13.5 ± 3.6	13.7 ± 3.7	13.6 ± 3.8	13.5 ± 3.6	13.4 ± 3.5	0.005
Creatinine, mg/dl	0.9 ± 0.2	0.9 ± 0.2	0.9 ± 0.3	0.9 ± 0.3	0.9 ± 0.2	0.052
Sodium, mmol/L	142.3 ± 1.9	142.3 ± 1.8	142.3 ± 1.9	142.3 ± 1.9	142.3 ± 1.9	0.748
Potassium, mmol/L	4.2 ± 0.3	4.2 ± 0.3	4.2 ± 0.3	4.1 ± 0.3	4.1 ± 0.3	<0.001
AST, U/L	23.6 ± 10.9	23.4 ± 11.6	23.4 ± 9.9	23.4 ± 9.4	24.3 ± 12.3	<0.001
ALT, U/L	25.7 ± 18.7	26.1 ± 21.4	25.8 ± 17.4	25.7 ± 17.4	25.4 ± 18.	0.136
Amylase, U/L	85.3 ± 29.3	83.1 ± 30.1	84.7 ± 29.5	85.5 ± 27.3	87.7 ± 29.9	<0.001
Total cholesterol, mg/dl	198 ± 33.8	196.3 ± 33.6	197.8 ± 33.5	199.1 ± 33.6	198.9 ± 34.6	<0.001
Triglyceride, mg/dl	113.8 ± 2.4	109.8 ± 78.9	117.4 ± 85.1	114.8 ± 82.6	113.2 ± 82.6	0.240
HDL‐C, mg/dl	54.2 ± 13.2	53.8 ± 13.2	53.6 ± 13	54.3 ± 13.3	54.9 ± 13.1	<0.001
LDL‐C, mg/dl	119.8 ± 30.5	119.1 ± 30.6	119.7 ± 30.4	120.6 ± 30.1	119.8 ± 30.7	0.201
CPK, U/L	119 ± 322	118 ± 222	122 ± 320	108 ± 140	126 ± 474	0.644
Hemoglobin, g/dL	14.5 ± 1.6	14.6 ± 1.5	14.5 ± 1.6	14.5 ± 1.6	14.3 ± 1.6	<0.001
Hematocrit, %	42.8 ± 4.1	43.2 ± 4.1	43 ± 4.1	42.8 ± 4.1	42.3 ± 4.1	<0.001
Pulmonary function test						
FEV1, %	98.1 ± 13.1	97.8 ± 13.2	97.6 ± 13.1	98.1 ± 13	98.9 ± 13.3	<0.001
FVC, %	92.6 ± 11.0	92.5 ± 10.9	92.3 ± 10.8	92.6 ± 11.1	93.0 ± 11.2	0.023
FEV1/FVC	82.1 ± 6.7	82.0 ± 6.8	82.0 ± 6.8	82.1 ± 6.5	82.2 ± 6.6	0.219
FEF 25%–75%	100.3 ± 28.3	100.6 ± 27.5	100.1 ± 27.7	100.4 ± 31.1	100.1 ± 27	0.585
Past medical history						
Hypertension, *n* (%)	1269 (10.8)	298 (10.9)	294 (10.3)	305 (10.5)	372 (11.4)	0.455
Dyslipidemia, *n* (%)	808 (6.9)	192 (7)	205 (7.2)	196 (6.7)	215 (6.6)	0.396
Asthma, *n* (%)	185 (1.6)	47 (1.7)	49 (1.7)	52 (1.8)	37 (1.1)	0.083
Family history of DM	4627 (29.7)	1226 (30.5)	1075 (29.2)	1075 (28.2)	1251 (31.2)	0.456

*Note*: Values are expressed as numbers (percentages) for categorical variables and mean ± standard deviation for continuous variables. *p* values were calculated using *t* test or correlation analysis for continuous variables and chi‐square test or Fisher exact test or Cochran–Armitage trend test for categorical variables.

Abbreviations: ALT, alanine aminotransferase, AST, aspartate aminotransferase; BMI, body mass index; BP, blood pressure; BUN, blood urea nitrogen; CPK, creatine phosphokinase; DM, diabetes mellitus; FEF, forced expiratory flow; FEV1, forced expiratory volume in 1 s; FVC, forced vital capacity; HbA1c, glycosylated hemoglobin; HDL‐C, high‐density lipoprotein cholesterol; LDL‐C, low‐density lipoprotein cholesterol; Q, quartile; TSH, thyroid‐stimulating hormone.

After 2 years, for each section, the rate of change of various variables was measured and compared (Table 2). As the baseline TSH level increased, ∆glucose decreased significantly after 2 years (*p* < 0.005), but ∆HbA1c did not show a significant difference between the groups (*p* = 0.487). ∆BMI (*p* < 0.001), ∆skeletal muscle mass (*p* < 0.001), and ∆body fat mass (*p* < 0.05) significantly increased with increasing baseline TSH levels. ∆Percent body fat (*p* = 0.306) and ∆waist circumference (*p* = 0.053) also increased, but this was not statistically significant. As the baseline TSH level increased, the ∆heart rate decreased (*p* < 0.001). In response to a rise in TSH, ∆FVC decreased (*p* < 0.05), and ∆FEV1/FVC showed a low tendency to decrease (*p* = 0.222).

**TABLE 2:** Change of various variables after 2 years according to baseline TSH

	Total	TSH_Q1	TSH_Q2	TSH_Q3	TSH_Q4	*p* for trend
	*n* = 15 557	*n* = 3712	*n* = 3823	*n* = 3870	*n* = 4152	
∆Glucose, mg/dl	2.3 ± 11.2	2.6 ± 12.4	2.5 ± 11.4	2.3 ± 10.8	1.9 ± 9.9	<0.005
Baseline	91.8 ± 11.4	91.5 ± 12.5	92 ± 11.3	91.9 ± 11.0	91.7 ± 10.9	0.659
2 YA	94.1 ± 12.2	94.4 ± 14.3	94.1 ± 11.6	94.2 ± 11.7	93.7 ± 11.2	0.024
∆HbA1c, %	0.1 ± 0.2	0.1 ± 0.0.2	0.04 ± 0.2	0.1 ± 0.2	0.1 ± 0.2	0.487
Baseline	5.4 ± 0.3	5.4 ± 0.3	5.4 ± 0.3	5.4 ± 0.3	5.4 ± 0.3	0.936
2 YA	5.5 ± 0.4	5.5 ± 0.4	5.5 ± 0.4	5.5 ± 0.4	5.5 ± 0.4	0.122
∆BMI, kg/m^2^	0.1 ± 1.1	0.01 ± 1.1	0.1 ± 1.0	0.1 ± 1.1	0.1 ± 1.0	<0.001
∆Skeletal muscle mass, kg	−0.02 ± 1.0	−0.1 ± 1.1	−0.008 ± 1.0	−0.01 ± 1.0	0.003 ± 1.0	<0.001
∆Body fat mass, kg	0.2 ± 2.4	0.2 ± 2.4	0.2 ± 2.7	0.2 ± 2.3	0.3 ± 2.3	<0.050
∆Percent body fat (%)	0.2 ± 2.7	0.2 ± 2.8	0.2 ± 2.7	0.3 ± 2.7	0.3 ± 2.7	0.306
∆Waist‐hip ratio	0.009 ± 0.60	0.004 ± 0.02	0.004 ± 0.02	0.02 ± 1.30	0.004 ± 0.02	0.665
∆Waist circumference, cm	0.5 ± 4.8	0.4 ± 4.8	0.5 ± 4.8	0.5 ± 4.7	0.6 ± 4.8	0.053
∆Heart rate, bpm	−0.4 ± 7.7	0.02 ± 8.1	−0.4 ± 7.7	−0.6 ± 7.4	−0.8 ± 7.6	<0.001
∆FEV1, %	−1.9 ± 15.9	−2.1 ± 7.9	−1.6 ± 18.4	−1.7 ± 18.1	−2 ± 16.5	0.993
∆FVC, %	−1 ± 6.6	−0.9 ± 6.4	−0.8 ± 6.6	−0.9 ± 6.6	−1.2 ± 6.8	0.046
∆FEV1/FVC	−0.4 ± 8.0	−0.5 ± 4.4	−0.5 ± 4.6	−0.5 ± 4.3	−0.3 ± 13.7	0.222
∆FEF 25%–75%	−0.2 ± 21.7	0.1 ± 24.1	−0.02 ± 27.3	−0.1 ± 17.1	−0.6 ± 17.2	0.172
Progression of DM, *n* (%)	377 (2.4)	98 (2.7)	87 (2.3)	103 (2.6)	89 (2.2)	0.243

*Note*: Values are expressed as numbers (percentages) for categorical variables and mean ± standard deviation for continuous variables. *p* values were calculated using *t* test or correlation analysis for continuous variables and chi‐square test or Fisher exact test or Cochran–Armitage trend test for categorical variables.

Abbreviations: BMI, body mass index; DM, diabetes mellitus; FEF, forced expiratory flow; FEV1, forced expiratory volume in 1 s; FVC, forced vital capacity; HbA1c, glycosylated hemoglobin; Q, quartile; TSH, thyroid‐stimulating hormone; YA, years after; ∆, rate of change.

Additionally, ∆ in each variable according to the change in TSH (∆TSH) during the two visits was evaluated (Table S1). ∆TSH_Q1 comprised 4.029 patients (∆TSH < −0.500), ∆TSH_Q2 comprised 3698 patients (−0.500 ≤ ∆TSH < 0.001), ∆TSH_Q3 comprised 3818 patients (0.001 ≤ ∆TSH < 0.530), and ∆TSH_Q4 comprised 4012 patients (0.530 ≤ ∆TSH). ∆Fasting blood glucose increased significantly as ∆TSH increased (all *p* < 0.005); however, ∆HbA1c showed no statistically significant difference (*p* = 0.095). An increased ∆TSH value resulted in a significant increase in ∆BMI (*p* < 0.001), ∆body fat mass, ∆percent body fat, and ∆waist circumference (all *p* < 0.001). The ∆FEV1/FVC ratio also showed a low tendency to decrease (*p* = 0.082).

Among the 15,557 patients, the incidence of diabetes mellitus after 2 years was 2.4% (377/15,557 patients). There was no statistically significant difference in the incidence of diabetes according to TSH_Q (*p* = 0.243) (Table 1). The incidence of diabetes tended to increase as ∆TSH increased, but the difference was not statistically significant (*p* = 0.131) (Table S1). The Pearson's correlation coefficient between ∆TSH and ∆HbA1c was −0.00077 (*p* = 0.924) (Figure S1A). Moreover, the Pearson's correlation coefficient between the absolute value of ∆TSH and the absolute value of ∆HbA1c was −0.00206 (*p* = 0.797) (Figure S1B).

Correlations were investigated with respect to the factors affecting the onset of diabetes (Table 3). There was no statistically significant association between baseline TSH levels and the incidence of diabetes after 2 years (OR 0.981; 95% Cl, 0.931–1.033; *p* = 0.460). When ∆TSH_Q4 was compared with ∆TSH_Q1, the incidence of diabetes was 1.126 times higher, but the difference was not statistically significant (OR 1.126; 95% Cl, 0.845–1.501; *p* = 0.418).

**TABLE 3:** Odds ratios for patients without diabetes at baseline who developed diabetes after 2 years

	Model 1		Model 2		Model 3	
	Odds ratio (95% CI)	*p* value	Odds ratio (95% CI)	*p* value	Odds ratio (95% CI)	*p* value
Baseline TSH	0.987 (0.936–1.041)	0.631	0.981 (0.931–1.033)	0.460		
TSH_Q1	Reference		Reference			
TSH_Q2	0.853 (0.637–1.142)	0.286	0.883 (0.658–1.186)	0.409		
TSH_Q3	0.970 (0.733–1.284)	0.833	0.995 (0.750–1.321)	0.974		
TSH_Q4	0.798 (0.597–1.067)	0.128	0.781 (0.582–1.048)	0.100		
**∆**TSH	1.008 (0.979–1.039)	0.579	1.002 (0.980–1.025)	0.868		
∆TSH_Q1	Reference		Reference			
∆TSH_Q2	0.969 (0.715–1.314)	0.841	0.939 (0.691–1.276)	0.687		
∆TSH_Q3	1.203 (0.903–1.602)	0.208	1.135 (0.850–1.516)	0.391		
∆TSH_Q4	1.177 (0.885–1.566)	0.261	1.126 (0.845–1.501)	0.418		
∆Glucose	1.076 (1.067–1.085)	<0.001	1.072 (1.063–1.081)	<0.001	1.049 (1.038–1.060)	<0.001
∆HbA1c	303.465 (196.543–468.552)	<0.001	297.199 (189.806–465.354)	<0.001	215.993 (132.385–352.406)	<0.001
∆BMI	1.012 (0.918–1.115)	0.818	1.061 (0.954–1.180)	0.277		
∆Skeletal muscle mass	0.779 (0.708–0.857)	<0.001	0.850 (0.771–0.936)	<0.001	0.734 (0.648–0.832)	<0.001
∆Body fat mass	1.054 (1.010–1.101)	<0.050	1.067 (1.019–1.118)	<0.010	0.957 (0.899–1.020)	0.178
∆Percent body fat	1.061 (1.021–1.102)	<0.005	1.064 (1.022–1.107)	<0.005	0.958 (0.891–1.031)	0.253
∆Waist‐hip ratio	0.973 (0.607–1.557)	0.908	0.985 (0.733–1.325)	0.921		
∆Waist circumference	0.985 (0.964–1.006)	0.154	0.989 (0.967–1.012)	0.335		
∆Systolic BP	1.002 (0.993–1.011)	0.698	1.002 (0.993–1.011)	0.648		
∆Diastolic BP	0.999 (0.986–1.012)	0.875	1.003 (0.990–1.016)	0.671		
∆Heart rate	1.019 (1.006–1.032)	<0.005	1.017 (1.004–1.031)	<0.010	0.998 (0.983–1.014)	0.833
∆Free T4	1.327 (0.859–2.049)	0.202	1.549 (0.999–2.400)	0.051		
∆BUN	1.003 (0.975–1.032)	0.829	1.001 (0.974–1.028)	0.966		
∆Creatinine	0.830 (0.327–2.107)	0.695	0.646 (0.255–1.636)	0.357		
∆Sodium	0.955 (0.910–1.001)	0.056	0.965 (0.920–1.013)	0.151		
∆Potassium	1.086 (0.798–1.479)	0.600	1.078 (0.792–1.469)	0.632		
∆FEV1	0.980 (0.965–0.995)	<0.010	0.995 (0.980–1.009)	0.471	1.001 (0.982–1.020)	0.918
∆FVC	1.000 (0.993–1.007)	0.993	1.000 (0.995–1.006)	0.930		
∆FEV1/FVC	1.003 (0.996–1.009)	0.435	1.003 (0.995–1.011)	0.531		
∆FEF 25%–75%	1.003 (0.999–1.006)	0.147	1.002 (0.998–1.006)	0.298		

*Note*: Odds ratios were calculated using logistic regression. Model 1: univariate. Model 2: adjusted by age (visit 2), sex (visit 2) + each variable. Model 3: adjusted by univariate < 0.05.

Abbreviations: BMI, body mass index; BP, blood pressure; BUN, blood urea nitrogen; CI, confidence interval; FEF, forced expiratory flow; FEV1, forced expiratory volume in 1 s; FVC, forced vital capacity; HbA1c, glycosylated hemoglobin; Q, quartile; TSH, thyroid‐stimulating hormone; ∆, rate of change.

∆glucose (OR 1.049; 95% Cl, 1.038–1.060; *p* < 0.001) and ∆HbA1c (OR 215.993; 95% Cl, 132.385–352.406; *p* < 0.001) were significantly associated with the development of diabetes. As skeletal muscle mass increased, the incidence of diabetes decreased significantly by 26.6% (OR 0.734; 95% Cl, 0.648–0.832; *p* < 0.001).

## 4 DISCUSSION

In hypothyroidism, glucose absorption in the intestine and gluconeogenesis in the liver is reduced.^14^, ^15^ Therefore, patients with diabetes and hypothyroidism are reported to have low fasting blood sugar levels. However, there are conflicting reports that hypothyroidism may increase glucose levels by increasing insulin resistance.^16^ In this ambiguity of evidence, it was not easy to see a relationship between ∆TSH and ∆glucose in the euthyroid state, but not in hypothyroidism.

Several studies have reported changes in blood glucose levels in subclinical hypothyroidism, except in the euthyroid state. It has been reported that there is a relationship between subclinical hypothyroidism and insulin resistance,^17^ and type 2 diabetes mellitus patients with subclinical hypothyroidism are vulnerable to glycemic control. Therefore, it has been argued that glycemic control improves after thyroid hormone treatment.^18^ However, this study did not find any significant differences in HbA1c levels or changes in fasting blood glucose according to baseline TSH levels in the euthyroid state. As TSH_Q increased, ∆blood glucose after 2 years significantly decreased, but there was no difference in ∆HbA1c or diabetes incidence. There was no significant difference in TSH_Q in the euthyroid state, but not in subclinical hypothyroidism. In this study, the difference in the ranges of TSH_Q (TSH boundary 1.184, 1.740, 2.552) was not clinically large.

In this study, when TSH levels increased in the euthyroid state, skeletal muscle mass decreased, and BMI, body fat mass, percent body fat, and waist‐hip ratio increased significantly. It is well known that body weight and body fat percentages increase in hypothyroidism,^19^ and muscle weakness occurs in subclinical hypothyroidism.^20^, ^21^ Although the mechanism is not yet clear; it is known to be due to decreased thermogenesis and fat tissue metabolism.^22^ It is also known that in hypothyroidism, insulin resistance increases, regardless of BMI.^23^, ^24^ However, in this study, the decrease in muscle and increase in fat when the TSH level increased, even in the euthyroid state, indicates that it is necessary to pay attention to muscle and fat management.

In fact, these changes in baseline body information, such as muscle and fat, may eventually contribute to an increase in blood glucose levels and the development of diabetes. Patients with type 2 diabetes have been reported to have more subclinical hypothyroidism than the general population.^8^ This is because thyroid diseases affect glucose metabolism. Insulin resistance is known to be induced in hypothyroidism and subclinical hypothyroidism.^17^, ^25^ Thus, patients with hypothyroidism are at a higher risk of diabetes.^26^ In patients with prediabetes, decreased thyroid function has been associated with decreased insulin sensitivity.^11^ Finally, patients diagnosed with high TSH levels in the euthyroid state, even in the prediabetes stage, have shown a 44% higher risk of developing diabetes.^11^ Therefore, a thyroid test is recommended for patients with prediabetes. Although it was not a direct study of blood glucose in the euthyroid state, changes in thyroid function were related to changes in body weight,^27^ and TSH and BMI were positively correlated.^27^ However, this study of euthyroid patients did not show a significant difference in the progression of diabetes mellitus according to ∆TSH for 2 years. This appears to be due to the short follow‐up period of 2 years. In the future, there is a need to check for the occurrence of diabetes at longer intervals.

We found that the higher the TSH level, the lower the proportion of men was observed; it is well known that thyroid disease is more common in women than in men.^28^, ^29^ Subclinical hypothyroidism, along with hypothyroidism, has been medically evaluated as a risk factor for cardiovascular diseases.^30^ In this study, in the euthyroid state, the lower the TSH level, the higher the heart rate, and the tendency to increase significantly even after 2 years. However, further research on this topic is required. Subclinical hypothyroidism has also been shown to affect the respiratory system and is associated with decreased vital lung capacity, anaerobic thresholds, and oxygen uptake.^31^ However, in this study, lung function did not show a significant correlation between TSH or ∆TSH and FEV1/FVC ratio. Whether this is a characteristic of the euthyroid state or is because of the short study period needs to be confirmed later.

In this study, the factors depicting the greatest association with diabetes development were glucose, HbA1c, and skeletal muscle. A negative correlation between lung function and HbA1c has been confirmed, and it is known that lung function affects the development of diabetes.^32^ However, it was difficult to identify clinically meaningful differences due to the short period of 2 years. FEV1, FVC, and FEF 25%–75% tended to increase as height increased in the same age group.^33^ This likely induced a relatively low BMI, and it is estimated that it may have lowered the risk of diabetes. However, it was difficult to obtain clinically meaningful results owing to the study's limitations over a short period.

This study had certain limitations. First, due to the short period of 2 years for viewing ∆TSH and ∆glucose, it was impossible to confirm the difference in the incidence of diabetes. More meaningful inferences could have been drawn if the study had been conducted for longer than 2 years. However, by observing changes in body composition that affected glucose levels in the euthyroid state, it was possible to estimate future increases in glucose levels. In the future, a large‐scale prospective study is needed to supplement these research results. Second, the average age of the patients was too low to confirm the occurrence of diabetes. However, in a study conducted in euthyroid obese children and adolescents, an increase in TSH level was highly associated with impaired glucose metabolism^34^; therefore, the results from this study are considered to be of sufficient value. Third, in this study, it was difficult to identify a clear causal relationship due to the nature of the retrospective cohort study,^35^, ^36^ and since various confounding variables could not be excluded, only correlations could be estimated. However, because this study used data from medical examinations at university hospitals, it contained many variables.^37^ Lastly, the medical check‐up did not include postprandial blood glucose measurement, one of the main diagnostic criteria for diabetes. Therefore, patients with impaired glucose tolerance may have been missed. However, we tried to minimize this possibility by including all patients' questionnaires, verifying their medication, and measuring fasting glucose or HbA1c levels.

To the best of our knowledge, this is the first study to identify changes in body composition according to baseline TSH and ∆TSH levels in euthyroid patients. In this study, although the association of blood glucose levels with the baseline TSH level in the euthyroid state was not statistically proven, owing to the short follow‐up period, a positive correlation between ∆TSH and ∆glucose was established. In addition, differences in various body compositions, such as a significant decrease in skeletal muscle mass and an increase in BMI and body fat mass according to the baseline TSH level, were demonstrated. Therefore, in patients with high TSH levels in the euthyroid state, attention and education to improve physical functions, such as increasing muscle mass and decreasing fat and weight loss, are required. Thus, based on the value of this study, a prospective long‐term follow‐up study with a larger number of patients should be conducted to explain a clear causal relationship.

## FUNDING INFORMATION

This study was supported by Daewoong Pharmaceutical.

## CONFLICT OF INTEREST

No potential conflict of interest relevant to this article is reported.

## Supporting information


**Figure S1** (A). Correlation graph between ∆TSH and ∆HbA1c. (B). Correlation graph between ∆TSH absolute value and ∆HbA1c absolute value
∆TSH: rate of change in thyroid‐stimulating hormone, ∆HbA1c: rate of change in glycated hemoglobin

**Table S1** Changes in various variables according to the difference in TSH (∆TSH) values between baseline and 2 years (divided by quartiles)
