# Supplementary figures and images for: Retrospective cohort analysis comparing changes in blood glucose level and body composition according to changes in thyroid‐stimulating hormone level
Source: J Diabetes. 2022 Sep 16;14(9):620–9. doi: 10.1111/1753-0407.13315 (PMC9512769; doi:10.1111/1753-0407.13315)

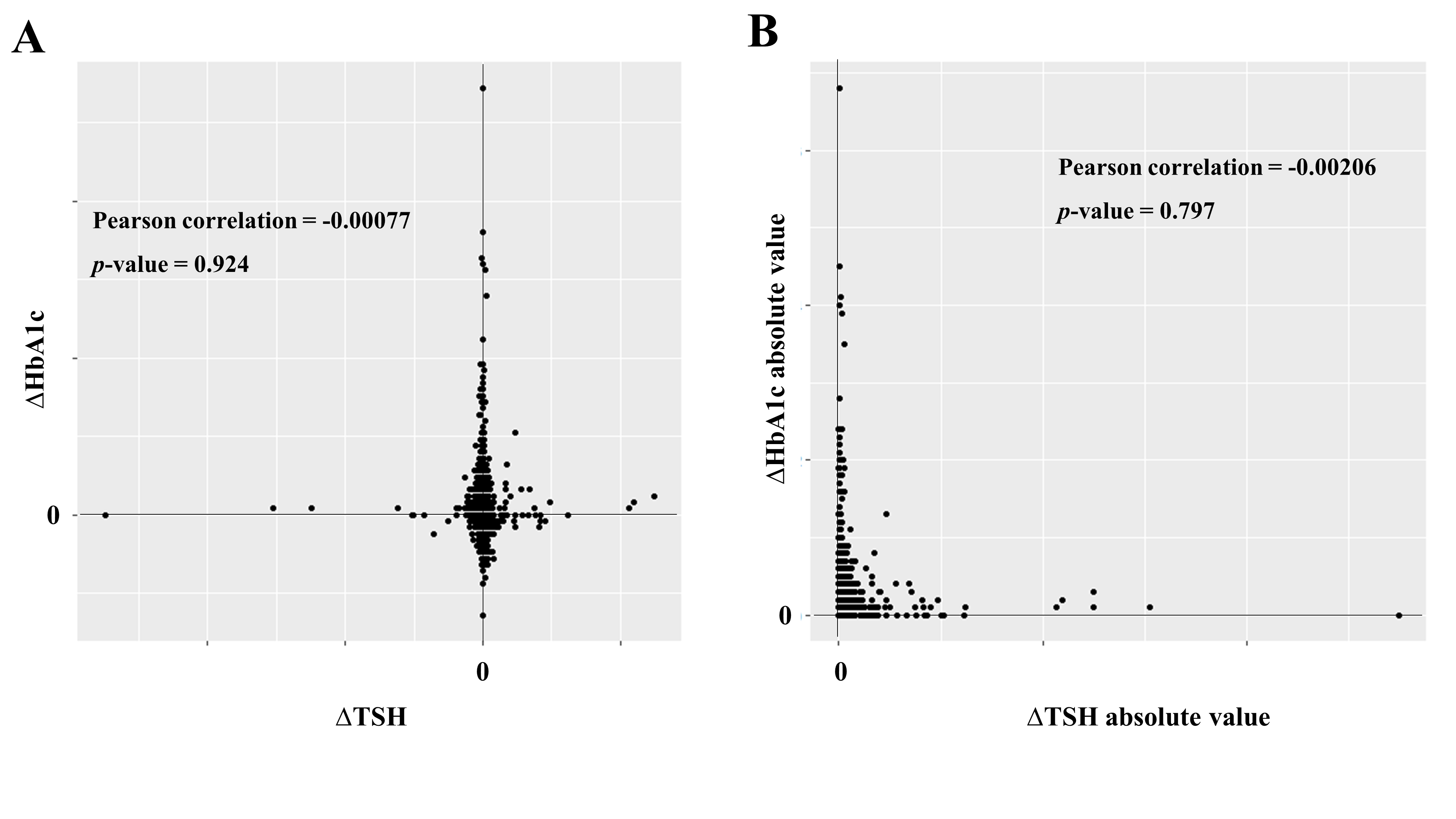

Supplement: Supplementary file 1 — Figure S1 (A). Correlation graph between ∆TSH and ∆HbA1c. (B). Correlation graph between ∆TSH absolute value and ∆HbA1c absolute value ∆TSH: rate of change in thyroid‐stimulating hormone, ∆HbA1c: rate of change in glycated hemoglobin [file JDB-14-620-s002.tif]
