# Supplementary material for: Retrospective cohort analysis comparing changes in blood glucose level and body composition according to changes in thyroid‐stimulating hormone level
Source: J Diabetes. 2022 Sep 16;14(9):620–9. doi: 10.1111/1753-0407.13315 (PMC9512769; doi:10.1111/1753-0407.13315)
Supplement: Supplementary file 2 — Table S1 Changes in various variables according to the difference in TSH (∆TSH) values between baseline and 2 years (divided by quartiles) [file JDB-14-620-s001.docx]

**Supplementary Table 1.** Changes in various variables according to the difference in TSH (∆TSH) values ​​between baseline and 2 years (divided by quartiles)

|  | **Total** | **∆TSH_Q1** | **∆TSH_Q2** | **∆TSH_Q3** | **∆TSH_Q4** | ***P* for trend** |
| --- | --- | --- | --- | --- | --- | --- |
|  | n=15557 | n=4029 | n=3698 | n=3818 | n=4012 |  |
| **∆Glucose mg/dL** | 2.3±11.2 | 1.9±10.8 | 2.1±11.4 | 2.6±10.4 | 2.7±11.9 | <0.001 |
| **Baseline** | 91.8±11.4 | 91.6±10.9 | 92.3±11.1 | 91.9±11.1 | 91.4±12.5 | 0.153 |
| **2 YA** | 94.1±12.2 | 93.5±12.3 | 94.3±12.6 | 94.4±12.3 | 94.1±11.7 | 0.041 |
| **∆HbA1c, %** | 0.1±0.2 | 0.1±0.2 | 0.1±0.3 | 0.1±0.2 | 0.04±0.2 | 0.095 |
| **Baseline** | 5.4±0.3 | 5.4±0.3 | 5.4±0.3 | 5.4±0.3 | 5.4±0.3 | 0.161 |
| **2 YA** | 5.5±0.4 | 5.5±0.4 | 5.5±0.4 | 5.5±0.4 | 5.5±0.4 | 0.889 |
|  |  |  |  |  |  |  |
| **∆BMI, kg/m^2^** | 0.1±1.1 | -0.009±1.2 | 0.03±1.0 | 0.1±1.0 | 0.2±1.0 | <0.001 |
| **∆Skeletal muscle mass, kg** | -0.02±1 | -0.04±1.0 | -0.03±1.0 | -0.03±1.0 | 0.001±1.1 | 0.069 |
| **∆Body fat mass, kg** | 0.2±2.4 | 0.1±2.3 | 0.1±2.6 | 0.2±2.3 | 0.4±2.4 | <0.001 |
| **∆Percent body fat, %** | 0.2±2.7 | 0.1±2.7 | 0.2±2.6 | 0.3±2.7 | 0.4±2.7 | <0.001 |
| **∆Waist-hip ratio** | 0.009±0.60 | 0.02±1.30 | 0.004±.0.02 | 0.004±0.02 | 0.005±0.02 | 0.221 |
| **∆Waist circumferences, cm** | 0.5±4.8 | 0.2±4.8 | 0.5±4.7 | 0.6±4.8 | 0.7±4.8 | <0.001 |
|  |  |  |  |  |  |  |
| **∆Heart rate, bpm** | -0.4±7.7 | 0.2±7.8 | -0.2±7.7 | -0.6±7.4 | -1.0±7.9 | <0.001 |
| **∆FEV1, %** | -1.9±15.9 | -2±16.7 | -2±7.8 | -1.9±18.4 | -1.6±18.0 | 0.157 |
| **∆FVC, %** | -1±6.6 | -0.9±6.7 | -0.9±6.3 | -1.1±6.8 | -0.9±6.7 | 0.676 |
| **∆FEV1/FVC** | -0.4±8.0 | -0.6±4.5 | -0.4±4.3 | -0.5±4.5 | -0.2±13.9 | 0.082 |
| **∆FEF 25-75%** | -0.2±21.7 | -0.2±31.2 | 0.1±16.7 | -0.3±17.4 | -0.1±17.7 | 0.920 |
|  |  |  |  |  |  |  |
| **Progression of DM, *n* (%)** | 377 (2.4) | 90 (2.2) | 80 (2.2) | 102 (2.7) | 105 (2.6) | 0.131 |

The p-value of the variable related to the rate of change was obtained from the data of visit two.

Values are expressed as numbers (percentages) for categorical variables and means ± standard deviation for continuous variables.

p-values were calculated using t-test or correlation analysis for continuous variables and chi-square test or Fisher's exact test† or Cochran-Armitage trend test for categorical variables.

TSH: thyroid-stimulating hormone; ∆: rate of change; YA: years after; HbA1c: glycated hemoglobin; BMI: body mass index; FEV1: forced expiratory volume in 1 s; FVC: forced vital capacity; FEF: forced expiratory flow; PEF: peak expiratory flow; DM: diabetes mellitus
